# The Characteristics and Mortality of Chinese Herbal Medicine Users among Newly Diagnosed Inoperable Huge Hepatocellular Carcinoma (≥10 cm) Patients: A Retrospective Cohort Study with Exploration of Core Herbs

**DOI:** 10.3390/ijerph191912480

**Published:** 2022-09-30

**Authors:** Shu-Ling Chen, Chia-Ying Ho, Wei-Chun Lin, Chao-Wei Lee, Yu-Chun Chen, Jiun-Liang Chen, Hsing-Yu Chen

**Affiliations:** 1 Division of Chinese Internal and Pediatric Medicine, Center for Traditional Chinese Medicine, Chang Gung Memorial Hospital, Taoyuan Branch, Taoyuan 333, Taiwan; 2 Department of Emergency Medicine, Chang Gung Memorial Hospital, Linkou Branch, Taoyuan 333, Taiwan; 3 Division of General Surgery, Department of Surgery, Linkou Chang Gung Memorial Hospital, Taoyuan 333, Taiwan; 4 College of Medicine, Chang Gung University, Guishan, Taoyuan 333, Taiwan; 5 Department of Family Medicine, Taipei Veterans General Hospital, Taipei 112, Taiwan; 6 School of Medicine, National Yang Ming Chiao Tung University, Taipei 112, Taiwan; 7 Institute of Hospital and Health Care Administration, National Yang-Ming University, Taipei 112, Taiwan; 8 School of Traditional Chinese Medicine, College of Medicine, Chang Gung University, Taoyuan 333, Taiwan; 9 Graduate Institute of Clinical Medical Sciences, College of Medicine, Chang Gung University, Taoyuan 333, Taiwan

**Keywords:** Chinese herbal medicine, network analysis, inoperable, huge hepatocellular carcinoma, survival analysis

## Abstract

For patients with inoperable huge hepatocellular carcinoma (H-HCC, tumor size ≥10 cm), treatment options are limited. This study aimed to evaluate the characteristics and outcomes of patients with H-HCC who use Chinese herbal medicine (CHM). Multi-institutional cohort data were obtained from the Chang Gung Research Database (CGRD) between 1 January 2002 and 31 December 2018. All patients were followed up for 3 years or until the occurrence of death. Characteristics of CHM users and risk of all-cause mortality were assessed, and core CHMs with potential pharmacologic pathways were explored. Among 1618 patients, clinical features of CHM users (88) and nonusers (1530) were similar except for lower serum α-fetoprotein (AFP) and higher serum albumin levels in CHM users. CHM users had significantly higher 3 year overall survival rates (15.0% vs. 9.7%) and 3 year liver-specific survival rates (13.4% vs. 10.7%), about 3 months longer median survival time, and lower risk of all-cause mortality. Core CHMs were discovered from the prescriptions, including *Hedyotis diffusa* Willd combined with *Scutellaria barbata* D.Don, *Salvia miltiorrhiza* Bunge., *Curcuma longa* L., *Rheum palmatum* L., and *Astragalus mongholicus* Bunge. CHM use appears safe and is possibly beneficial for inoperable H-HCC patients; however, further clinical trials are still required.

## 1. Introduction

Liver cancer is the second-most common cause of cancer-related death worldwide and is one of the few neoplasms with a steadily increasing incidence and mortality. Hepatocellular carcinoma (HCC) makes up approximately 90% of all cases of primary liver cancer and is the fourth most common cause of cancer-related death worldwide [1,2,3,4]. The illness is usually associated with chronic liver immune disorders caused by excessive alcohol consumption, hepatitis B virus (HBV) infection, and hepatitis C virus (HCV) infection [5]. The international guidelines for treatment remain controversial for patients with huge HCC (H-HCC, tumor size ≥10cm), because these patients not only have a high recurrence rate after resection but often exhibit poor tumor differentiation, vascular invasion, and satellite nodules [6,7,8]. Our previous study showed the superiority of hepatectomy, where feasible, for H-HCC in survival benefits; however, the prognosis of inoperable H-HCC was poor [9]. Several alternative treatment modalities other than hepatectomy or transplantation for HCC have emerged, including transarterial chemoembolization (TACE), radiofrequency ablation (RFA), stereotactic body radiotherapy (SBRT), percutaneous ethanol injection (PEI), and target therapy, each with advantages and disadvantages. For example, local control rates with TACE are poor, RFA efficacy is limited by the proximity of nearby structures and has significantly worse freedom of local progression for tumors ≥2 cm when compared to SBRT, and SBRT demonstrated no significant survival benefit when compared to RFA in HCC patients [10,11,12,13]. Although novel pharmacological therapies focusing on cell-cycle regulation, DNA repair, and immune modulation have been studied, their application in clinical practice is still not yet feasible [14,15,16,17].

Chinese herbal medicine (CHM) has recently been considered a viable option for treating HCC because of its multitarget and coordinated intervention effects [18]. The extensive practice of phytochemical and molecular biological treatment in CHM-based compounds has shown prospects for anti-HCC products [19]. CHM can inhibit the growth of HCC cells, both in vitro and in vivo, by inducing cell death and immunoregulation, inhibiting metastasis, reducing the inflammatory response, and increasing antiviral activity [20]. In recent years, several retrospective cohort studies have also shown that combined CHM therapy can improve the survival rate of patients with unresectable HCC after TACE and RFA [21,22] in addition to all HCC patients [23]; nonetheless, the outcome of patients with unresectable HCC may differ because of the tumor size, which has been less assessed. Liu et al. reported that long-term (>90 days) use of adjuvant CHM may prolong the median survival time and reduce the mortality of HCC patients regardless of etiology, tumor stage, liver function level, or type of initial treatment [24]. However, there are limited data in the literature regarding the impact of CHM on patients with inoperable H-HCC (≥10 cm), whether about their therapeutic outcome, survival, or prescription analysis under treatment of alternative procedures coupled with CHM [25,26,27].

This multi-institutional cohort study aimed to evaluate the prognosis of H-HCC patients with CHM use, exemplify CHM prescription analysis, and explore the core CHMs and their possible pharmacological mechanisms for H-HCC (≥10 cm).

## 2. Material and Methods

### 2.1. Data Source

Eligible HCC patients were identified from the Chang Gung Research Database (CGRD), which contains the original electronic medical records from the Chang Gung Memorial Hospital (CGMH). As the most extensive private hospital system in Taiwan, CGMH comprises nine medical institutes with branches in Keelung, Tucheng, Taipei, Linkou, Taoyuan, Yunlin, Chiayi, Kaohsiung, and Fengshan. The CGRD covers about 20% of Taiwan’s population of cancer patients, with outpatient coverage rates of up to 34%; it is, therefore, a credible target database for clinical studies [28]. As the largest multi-institutional electronic medical record (EMR) collection in Taiwan, the CGRD contains detailed information, including gender, age, diagnosis of each outpatient/emergency visit or admission, medications, comorbidities, procedures, nursing care, national health insurance payments, laboratory data, and cancer registry [29]. In addition, the cancer registry contains detailed information including diagnosis date, cancer stage, tumor size, dates of every treatment modality, date and type of recurrence, and date of death [30]. 

The CGRD also includes detailed CHM prescription records of CGMH patients. The CHMs can be classified into two groups according to their components: single herb (SH) and herbal formula (HF). SH refers to a single material, for instance, *Hedyotis diffusa* Willd or *Scutellaria barbata* D.Don., while HF is composed of multiple SHs recorded in classical use of traditional Chinese medicine (TCM), for example, Shen-Ling-Bai-Zhu-San. Before marketing, HFs are manufactured and mixed in proportions regulated by classical TCM theory. Accordingly, TCM doctors may prescribe HFs or SHs on the basis of each patient’s condition. All CHMs are made by pharmacies following “good manufacturing practices” and have zero-tolerance regulations for potential renal or liver toxicities and pollution by pesticide or heavy metals.

### 2.2. Study Population and Ethical Considerations

**Figure 1:**
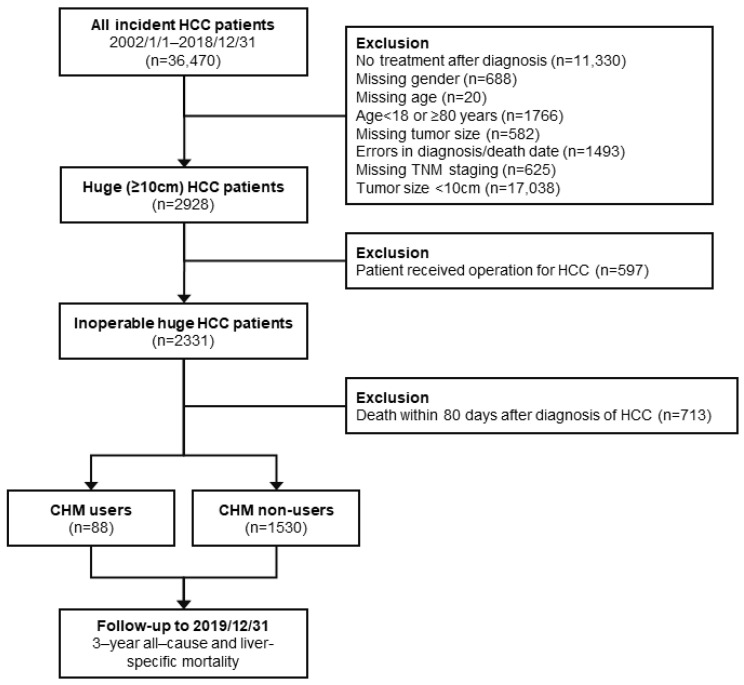
Flow diagram of this study.

Figure 1 shows a flow diagram of this study. First, we retrieved patients newly diagnosed with HCC (ICD-10-CM code: C22.0, ICD-9-CM code: 155) between 1 January 2002 and 31 December 2018 from the CGRD. Next, we excluded patients with errors in diagnosis or death date, patients with missing gender, age, tumor size records, or TNM staging, and patients who received no treatment after diagnosis. We also excluded patients with tumor size <10 cm, those who received surgery for HCC, those who were aged ≥18 and <80 years, and those who died within 80 days of diagnosis. Then, we categorized the remaining eligible subjects into two groups as a function of whether CHM treatment was used after diagnosis. CHM users were defined as the population receiving at least two CHM treatments during the study period, while those who did not receive CHM treatment were classified as CHM nonusers. The intention-to-treat design was applied to define CHM users and nonusers; therefore, patients were not reallocated between groups during follow-up. The entire study protocol was approved by the Institutional Review Board of the Chang Gung Medical Foundation in Taiwan (IRB No.: 201900800B0). Written informed consent was exempted because the identification number of each patient was encrypted, and it was impossible to deduce the identity of the patients.

### 2.3. Outcome Assessment

All eligible subjects were followed up until the occurrence of the primary endpoint, which was set at a maximum of 3 years after diagnosis, or until the end of 2019 (Figure 1). The primary outcome of this study was the overall survival rate (OS), in which death could be caused by any causes, and the survival time was calculated from the date of diagnosis to the date of death. The secondary outcome of this study was the disease-specific survival rate, which was referred to as liver-specific survival rate and was only applied to patients who died due to HCC-related causes.

### 2.4. Study Covariates

Demographic covariates, including gender, age, comorbidities, and lifestyles, were obtained from the CGRD. HCC-related covariates were also retrieved, such as the Child–Pugh score classification, cirrhosis, cancer staging according to both TNM staging and Barcelona Clinic Liver Cancer (BCLC) classification, tumor size, and initial treatment modalities (including TACE, RFA/PEI, target therapy, and chemotherapy). In addition, biochemical profiles were recorded to establish patients’ baseline physical condition, including α-fetoprotein, serum albumin, hemoglobin, platelet, international normalized ratio (INR), aspartate aminotransferase (AST), alanine aminotransferase (ALT), and total bilirubin. The laboratory values for these biochemical profiles were obtained within 1 year before diagnosis, and the value closest to the diagnosis date was used. For the regression models, the laboratory values were reclassified to binary covariates as ‘normal’ and ‘abnormal’ according to the reference values. In addition, two outpatient visits or one inpatient record of hypertension, type 2 diabetes mellitus (DM), chronic hepatitis (HBV, HCV, or HBV + HCV), or chronic kidney disease (CKD) within 1 year before the diagnosis of HCC were considered comorbidities. The diagnosis codes used in this study are listed in Supplementary Material S1. The use of metformin, aspirin, anti-HBC/HCV therapy, and diuretics more than 30 days was also recorded because they may influence the prognosis of HCC [31,32,33,34].

### 2.5. Bias Assessment

To minimize the confounding bias, we matched CHM users and nonusers through different propensity score (PS)-based models for the sensitivity tests in this study. Furthermore, the use of the CGRD prevents possible recall bias because it is an electronic medical record that is updated simultaneously during daily clinical practice in the CGMH. Since there was no exact time of CHM treatment initiation for HCC patients given in the clinical guideline, immortal time bias may be present; hence, we excluded patients who died within 80 days during the follow-up period, which was determined by calculating the median time between the date of diagnosis and receiving CHM treatment. In addition, all CGRD entries were linked to the national death registry database supported by the National Health Informatics Project, which allowed us to trace patients’ outcomes afterward, even if the patient died outside the CGMH. As a result, we could rule out the possible biases in registration and detection of the cause of death in the CGRD.

### 2.6. Statistical Analysis

Outcome assessment and Chinese herbal medicine network (CMN) analysis on prescriptions made for inoperable H-HCC were performed in this study. Baseline demographic features were presented as the median with interquartile range (IQR) for continuous covariates or as counts with percentages for categorical covariates. The differences in demographics between CHM users and nonusers were examined with Student’s *t*-tests and chi-square statistics. Moreover, the characteristics of CHM users were assessed by logistic regression when considering all possible covariates. The OS and liver-specific survival were estimated using the Kaplan-Meier method at 1, 2, and 3 year timepoints. The risk of all-cause mortality was assessed using the Cox regression model. Adjusted hazard ratios (aHR) were calculated by considering the imbalanced demographic covariates between CHM users and nonusers. Furthermore, we used several PS-based models to correct for the imbalanced case numbers and estimated the risk of mortality under different model settings, including overlap weighting, average treatment effect on the treated (ATT), and inverse probability treatment weighting (IPTW). For example, the overlap weighting model added weights to subjects on the basis of demographic features between CHM users and nonusers, including age, gender, lifestyles, comorbidities, initial treatments, and medications [35]. These covariates were used to generate the probability of using CHM as PS, and the PS was assigned as a weight to CHM nonusers while 1 − PS was assigned to CHM users [36]. In addition, covariates other than those used to generate PS were used in the Cox regression to assess the risk of all-cause mortality in different PS-based models. As a different sampling method, all subjects without a landmark setting and those with a 120 day landmark setting were also used to examine the risk of all-cause mortality. We also performed multivariate Cox regression stratified by demographic covariates to confirm the associations between using CHM and the OS in different subgroups of subjects.

Furthermore, with CMN analysis, we graphically demonstrate the treatment principle and the core CHM used for H-HCC. The process of CMN build-up was reported in detail in our previous studies [37]. Association rule mining (ARM) was used to determine the common CHM combinations and social network analysis (SNA) was used to graph and analyze the CMN. We collected commonly used CHM into clusters according to their relationships and identified core CHMs that had high prevalence and were highly connected to other CHMs (i.e., that were used when the core CHM was prescribed). According to TCM theory, the CHM was prescribed on the basis of the individual syndrome, and we identified each CHM cluster and its core CHM by integrating SNA and CHM indications from the CHM pharmacopoeia. We also analyzed possible molecular mechanisms of the core CHM from the CMN. To summarize the molecular mechanisms of CHMs, the target proteins of each cluster were used to propose the possible molecular mechanisms using overrepresentation tests in REACTOME, which is a freely accessible web resource to estimate, interpret, and visualize the molecular mechanisms of a given group of genes or proteins [38]. To analyze the CMN in this study, Stata statistical software (release 16; StataCorp, College Station, TX, USA) and the network analysis software NodeXL were used to determine the core CHM in complex CMNs, with a significance threshold of *p* < 0.05.

## 3. Results

### 3.1. Features of CHM Users

The demographic and clinical data are presented in Table 1. A total of 1618 patients with H-HCC (100.0%), consisting of 252 (15.6%) females and 1366 (84.4%) males, were included in the study. Patients were 58.0 (49.0–68.0) years old, with age groups as follows: ≤40 years old, n = 167 (10.3%); 41–60 years old, n = 747 (46.2%); ≥61 years old, n = 704 (43.5%). The comorbidities included the following: diabetes, n = 288 (17.8%); hypertension, n = 434 (26.8%); chronic hepatitis HBV, n = 787 (48.6%); chronic hepatitis HCV, n = 186 (11.5%); chronic hepatitis HBV and HCV, n = 67 (4.1%). About 2.1% patients had a fatty liver when H-HCC was diagnosed. The lifestyle factors consisted of the following: cigarette smoking, n = 312 (19.3%); alcohol consumption, n = 253 (15.6%); betel nut chewing, n = 99 (6.1%). According to the Child–Pugh Score classification of disease severity, there were 658 patients (74.8%) in class A, 201 patients (22.8%) in class B, and 21 patients (2.4%) in class C. There were 651 (40.2%) patients with cirrhosis and 967 (59.8%) patients without cirrhosis. The tumors were 12.7 (11.0–15.0) cm in size. The TNM staging according to AJCC v. 8 consisted of 73 (4.5%) stage I patients, 30 (1.9%) stage II patients, 1057 (65.3%) stage III patients, and 458 (28.3%) stage IV patients. According to the BCLC classification, there were 194 (17.5%) patients in stage B, 834 (75.4%) patients in stage C, and 77 (7.0%) patients in stage D. For initial HCC treatment, 437 (27.0%) patients were treated with TACE, 20 (1.2%) patients were treated with RFA/PEI, 397 (24.5%) with target therapy, and 316 (19.5%) patients were treated with chemotherapy. Regarding medication, 71 (4.4%) patients were given anti-HCV/HBV therapy, 83 (5.1%) were on metformin, 71 (4.4%) were taking aspirin, and 129 (8.0%) had diuretic therapy. Biochemical profiles were summarized as follows: α-fetoprotein, 749.7 (26.1– 7359.5) ng/mL; albumin, 3.7 (3.3–4.1) g/dL; hemoglobin, 12.7 (11.1–14.2) g/dL; platelets, 224.0 (157.0–302.0) × 10^3^/µL; INR, 1.1 (1.1–1.2); AST, 85.0 (57.0–135.0) U/L; ALT, 51.0 (32.0–82.0) U/L; total bilirubin, 1.0 (0.7–1.4) mg/dL. 

A comparison of the above clinical variables between CHM users (n = 88) and CHM nonusers (n = 1530) is also given in Table 1. There were no significant differences in the variables observed between the two groups (all *p* > 0.05) except for albumin (*p* = 0.003); however, the value of albumin was still within the normal range in both groups (4.0 [3.5–4.2] for CHM users and 3.7 [3.2–4.1] for CHM nonusers). When considering all covariates, cigarette smoking (adjusted odds ratio [aOR] = 0.36; 95% CI = 0.14–0.90; *p* = 0.029) and high α-fetoprotein (> 400 ng/mL) (aOR = 0.41; 95% CI = 0.21–0.81; *p* = 0.010) were less associated with CHM use, while the occurrence of cirrhosis was positively associated with CHM use (aOR = 2.50; 95% CI = 1.14–5.47; *p* = 0.022) (Table 2). 

### 3.2. Overall Survival Analysis

**Figure 2:**
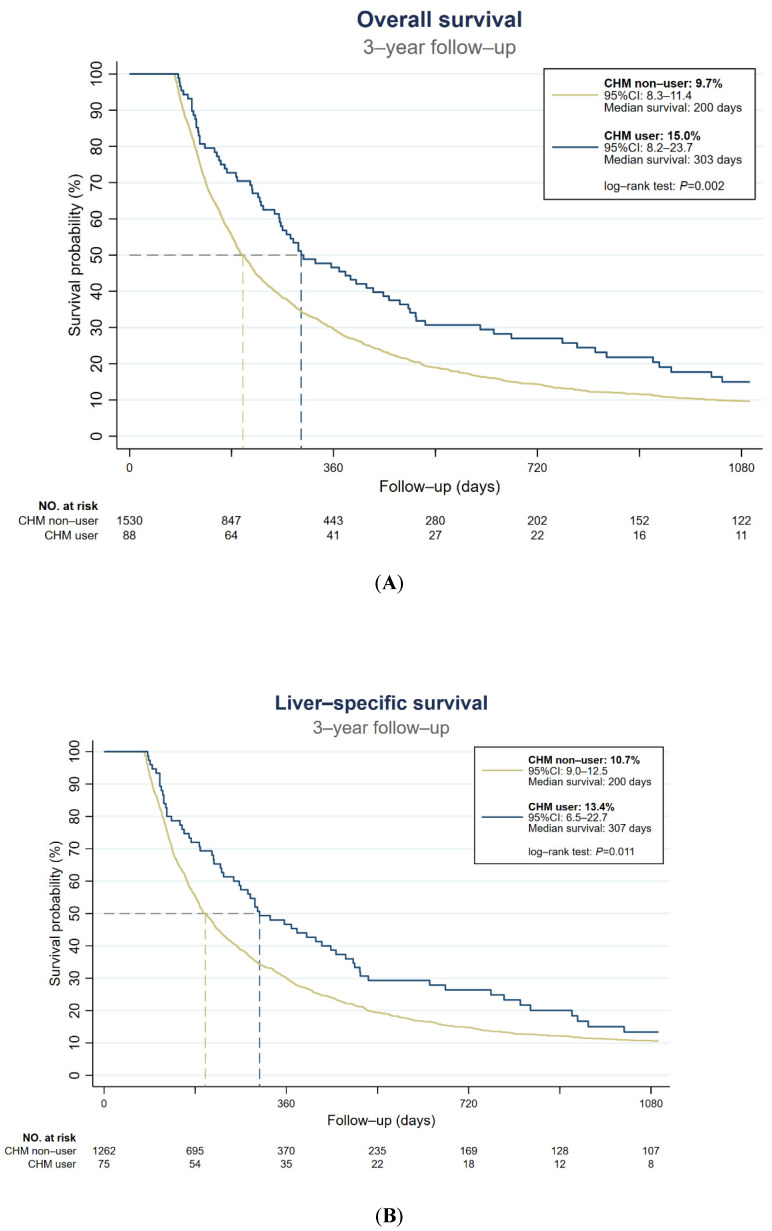
Kaplan–Meier estimation of (**A**) overall survival and (**B**) liver-specific survival of all inoperable H-HCC patients. The dotted lines present the median survival days.

Table 3 presents the outcome of inoperable H-HCC among all subjects. The current study found significant differences between CHM users and CHM nonusers at any of the 1, 2, or 3 year timepoints for OS and liver-specific survival rate (*p* < 0.05). CHM use was associated with higher OS at each timepoint. The favorable tendency for liver-specific survival could be seen when excluding subjects unrelated to H-HCC at each time point (Table 3). For CHM users, the risk of all-cause mortality was reduced by 31% compared with CHM nonusers (HR = 0.69; 95% CI = 0.56–0.85; *p* = 0.001, Table 4). When considering all accessible covariates, CHM users even had a 38% lower risk of all-cause mortality (aHR = 0.62; 95% CI = 0.44–0.87; *p* = 0.006). Further, CHM use had a duration-dependent tendency to reduce all-cause mortality. Table 4 demonstrates that the risk of all-cause mortality was significantly reduced among CHM users with accumulative duration >28 days (HR = 0.54; 95% CI = 0.40–0.74; *p* < 0.001; and aHR = 0.44; 95% CI = 0.26–0.74; *p* = 0.002; Table 4), but no significant difference was found between CHM users with duration <28 days (HR = 0.84; 95% CI = 0.63–1.10; *p =* 0.204, and aHR = 0.85; 95% CI = 0.58–1.25; *p* = 0.416). Figure 2A shows the Kaplan–Meier estimation of OS and liver-specific survival of all patients with inoperable H-HCC. At the 3 year follow-up, the OS was 9.7% (95% CI = 8.3–11.4) for CHM nonusers and 15.0% (95% CI = 8.2–23.7) for CHM users. The median survival for CHM nonusers and CHM users was 200 days and 303 days, respectively. The outcomes of CHM users were significantly superior to those of CHM nonusers (log-rank test, *p* = 0.002). The same tendency was found with the liver-specific survival (Figure 2B). Sensitivity tests with different PS-based matching methods and different populations were performed to remove potential confounding and selection biases (Table 5). The ATT, IPTW, and overlap weighting tests all presented consistent and significantly favorable HR. Significance differences between populations were observed on all subjects with a 120 day landmark design (HR = 0.70; 95% CI = 0.54–0.91; *p* =0.008, and aHR = 0.67; 95% CI 0.49–0.93; *p* = 0.016) and without landmark design (HR = 0.55; 95% CI = 0.44–0.69; *p* < 0.001; and aHR = 0.58; 95% CI = 0.44–0.76; *p* < 0.001). As for the subgroup analysis, patients with initial treatment (HR = 0.73; 95% CI = 0.54–0.98; *p* = 0.037, and aHR = 0.59; 95% CI = 0.40–0.88; *p* = 0.009) were revealed to have better HR compared to CHM nonusers. Additionally, higher disease severity, including low albumin, coexistence of cirrhosis, and high α-fetoprotein, led to a lower risk of all-cause mortality.

### 3.3. Prescription Analysis on CHM Used for H-HCC

**Figure 3:**
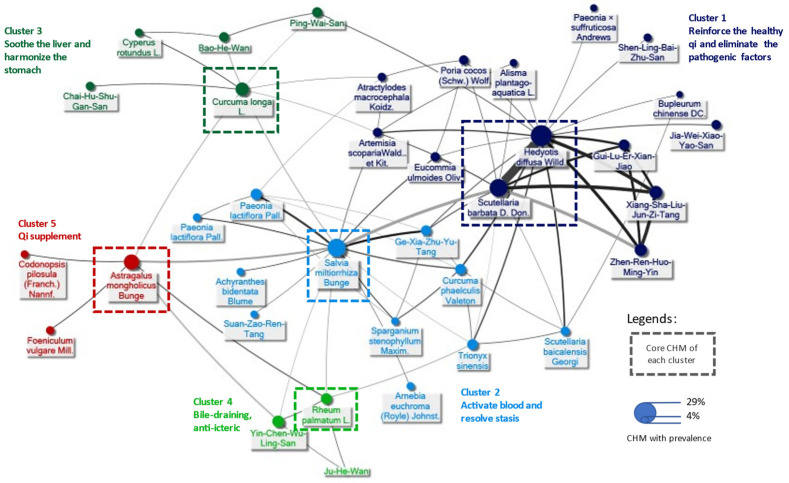
Demonstration of CHM prescriptions used for H-HCC as shown by the Chinese herbal medicine network (CMN). The core CHM of each cluster was identified by network analysis.

A total of 417 prescriptions were made from 293 CHMs, and 7.7 ± 4.4 (mean ± standard deviation) kinds of CHMs were used in each prescription. Table 6 lists the top 10 single CHMs. *Hedyotis diffusa* Willd. (43.2%) was the most used CHM for H-HCC, followed by *Salvia miltiorrhiza* Bunge. (31.7%). We also determined the 100 most prevalent CHM–CHM combinations used to construct CMN (Supplementary File S2). Figure 3 demonstrates the CHM network by clustering these combinations. This network presents a comprehensive overview of CHM for H-HCC, in which larger circles denote a higher prevalence of single CHM, and thicker and darker edges between CHMs represent more prevalent and stronger connections. By integrating SNA and CHM indications from the CHM pharmacopeia, five CHM clusters were identified, each with its own tendency to treat a specific TCM syndrome. The clusters are distinguished by different colors in Figure 3. The core CHMs among the five clusters could be identified from their relatively high prevalence and high connectivity to other CHM within clusters, which were calculated using SNA on the CMN. The composition of all herbal formulas (HFs) and the list of CHM and ingredients categorized by different CHM clusters in the CHM network are provided in Supplementary Materials S3 and S4. Cluster 1 reinforces healthy qi and eliminates the pathogenic factors, and has *Hedyotis diffusa* Willd and *Scutellaria barbata* D.Don as its core CHMs. HFs including Gui-Lu-Er-Xian Jiao, Xiang-Sha-Liu-Jun-Zi-Tang, and Zhen-Ren-Huo-Ming-Yin shared strong connections with the core CHMs in Cluster 1. Cluster 2 activates blood and resolves stasis, and has *Salvia miltiorrhiza* Bunge as its core CHM. Cluster 3 soothes the liver and harmonizes the stomach, and has *Curcuma longa* L as its core CHM. Cluster 4 focuses on bile-draining, as well as anti-icteric management, and has *Rheum palmatum* L. as its core CHM. Lastly, cluster 5 supplements qi, and has *Astragalus mongholicus* Bunge as its core CHM. 

### 3.4. Proposed Molecular Pathways of Core CHMs for H-HCC

**Figure 4:**
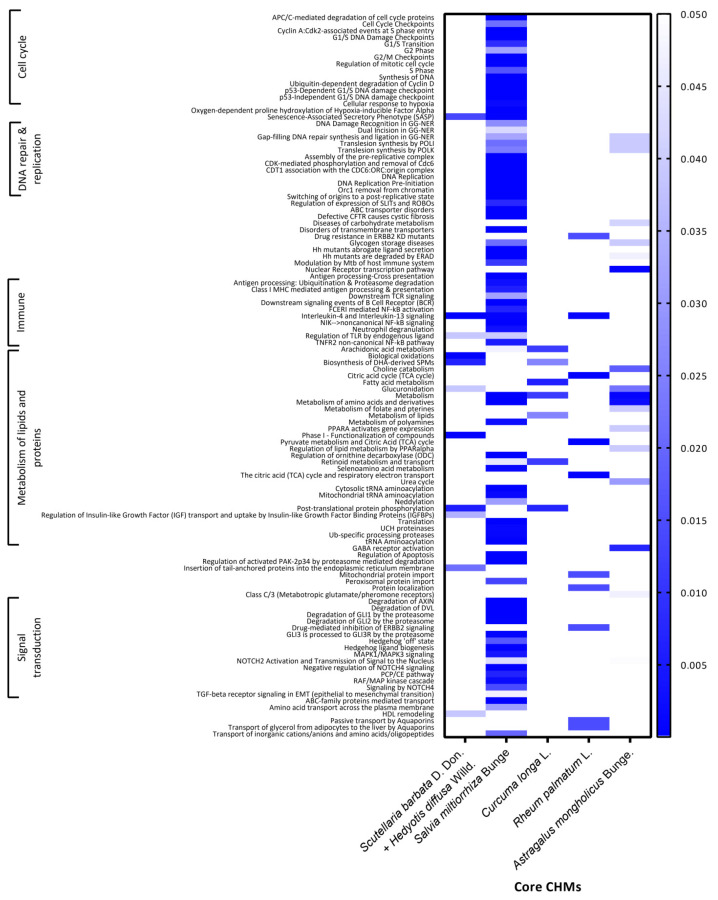
The molecular pathways covered by core Chinese herbal medicine (CHM) in the CHM network (CMN). These pathways were proposed by the REACTOME pathway database.

Furthermore, we investigated possible actions of core CHMs by consulting the REACTOME pathway database to explore the mixed pharmacological effects of core CHMs. A total of 37 kinds of CHM (with 124 ingredients) were used to find their target proteins. The list of total possible binding proteins of each CHM cluster is provided in Supplementary Materials S3. Figure 4 shows the molecular pathways covered by core CHMs in the CMN. These pathways included the cell cycle, DNA repair and replication, the immune system, metabolism of lipids and proteins, and signal transduction. Overall, we found 109 molecular pathways covered by five core CHMs in the network. Among the five CHM clusters, only Cluster 2 affected binding proteins across all five different pathways, and all five core CHMs affected pathways in the metabolism of lipids and proteins.

## 4. Discussion

Our study demonstrates the potential of using CHM for patients with inoperable H-HCC, with CHM users having better 1 and 2 year OS and liver-specific survival rates (Table 3), with a duration-dependent tendency (Table 4). Additionally, CHM use may significantly reduce all-cause mortality with or without considering all accessible covariates in the regression model (Table 4). We also performed sensitivity tests with different PS-based matching methods and other sampling populations (with 120 day landmark analysis, age, gender, and initial treatments) to reduce potential confounding and selection biases (Table 5). Since CHM is not yet a part of the standard management of HCC, the consistent results of sensitivity tests show the feasibility of using CHM among patients with inoperable H-HCC. Moreover, five core CHMs with different CHM indications and possible pharmacologic pathways were explored. 

According to the BCLC staging system, liver resection is the best treatment for patients with BCLC stage 0 and stage A diseases; however, treatment options are often limited for inoperable patients, especially when the tumor burden is heavy. For these patients, individualized treatments are often demanded, and we propose that CHM may be used at this stage. Patients with asymptomatic large HCCs (>5 cm) without major vascular invasion or extrahepatic spread are categorized as having intermediate stage disease (BCLC stage B) and should receive TACE [39,40]. According to Lee et al., surgery is superior to TACE for patients with H-HCC (≥10 cm) in terms of disease control and survival, and should be performed for single H-HCC or H-HCC with limited daughter nodules confined in the same lobule, for which the median survival time may reach nearly 52.6 months in surgery group [9]. Unfortunately, the prognosis was much worse for patients with inoperable H-HCC. For inoperable large HCC (≥5 cm, media tumor size 8.6 [7–12.3] cm), Yoon et al. reported that the median survival time dropped to 15.8 months with modified cisplatin-based TACE [41]. According to the Sorafenib Hepatocellular Carcinoma Assessment Randomized Protocol (SHARP) trial [42,43,44,45], target therapy with sorafenib extends the median survival of patients with advanced HCC to 10.7 months. However, the study by Cheng et al. [46] reported that the median survival of patients with advanced HCC dropped to 6.5 months when treated with sorafenib, which could be due to the large degree of variation in disease severity in the study population. Tang et al. reported that combined CHM use with TACE and RFA for inoperable patients (main mean tumor sizes of 5.6 cm and 6.8 cm, respectively) may significantly improve 3 year overall survival (37.74% and 38.3% respectively) [21,22]. Our subgroup analysis also supported that, even with a much larger primary tumor size (mean = 12.7 cm), CHM users with initial treatments including TACE, RFA/PEI, chemotherapy, and target therapy may have much improved overall survival (Table 5). For other prognostic covariates, such as hypoalbuminemia, coexistence of cirrhosis, and high α-fetoprotein, use of CHM tended to be associated with lower risk of 3 year all-cause mortality. As a result, we believe that combined CHM use may be a possible option for better overall survival for patients with inoperable H-HCC.

Previous studies showed that HCC patients with tumor size larger than 10 cm were generally younger and had less HCV infection when compared to HCC patients with smaller tumor sizes [9,47], which is consistent with our current study. Several studies noted that, compared with older HCC patients, younger HCC patients have lower positive rates of hepatitis C virus, lower proportion of cirrhosis, and higher frequency of increased AFP levels [48,49], suggesting possible differences in the carcinogenesis of HCC between young and elderly patients. Although the hepatitis B virus may be important in HCC development without associated liver cirrhosis, nonviral causes such as nonalcoholic fatty liver disease accounted for nearly 40% of H-HCC patients in the current study, which implies the importance of metabolic disorder in HCC. Likewise, REACTOME pathway analysis revealed that all five core CHMs affected pathways in the metabolism of lipids and proteins, which may also suggest possible mechanisms for the carcinogenesis of H-HCC, as well as the value of targeting these mechanisms to manage H-HCC. Further studies are warranted to confirm these associations. 

According to TCM theory, healthy qi not only promotes the metabolic function of each organ, but also defends the human body from pathogens. Solid cancers such as H-HCC may be viewed as a result of an internal pathogen caused by qi and blood stasis, such as dysregulation of the metabolic and immune systems [50,51]. This explains the core CHM of Cluster 2 (*Salvia miltiorrhiza* Bunge) and how activating blood and resolving stasis may a have beneficial effect on fatty liver, nonalcoholic fatty liver disease (NAFLD), and its progressive form, nonalcoholic steatohepatitis (NASH) [52]. The active constituent of *Salvia miltiorrhiza* Bunge, Tanshinone II-A (TIIA), was reported to have many beneficial effects on HCC, including arrest at G0/G1 phase, inducing apoptosis, as well as anti-invasion and anti-metastasis effects [53]. Tanshinone II-A can also protect against the hepatic lipid peroxidation process, and it may regulate intracellular molecular targets like PPARα, CYP1A2, and MMP2, to mediate lipid metabolism and promote antioxidant activity and anti-fibrogenesis [54,55]. *Curcuma longa* L (turmeric) is the only core CHM that affects only lipid and protein metabolism pathways, and its liver-protective and antioxidant features have been widely reported [56]. Curcumin is the main constituent reported to be effective against numerous types of oxidative-associated liver diseases [57,58,59]. Curcumin and curcumin-rich *Curcuma longa* L. extract have been reported to promote recovery in a representative model of carbon tetrachloride (CCl_4_)-induced hepatotoxicity for both acute and chronic hepatotoxicity and lipid dyslipidemia [60]. 

Pharmacological pathway analysis revealed that, in addition to affecting the metabolic pathways of lipids and proteins, the core CHMs of Cluster 1 (*Hedyotis diffusa* Willd and *Scutellaria barbata* D.Don) and Cluster 4 (*Rheum palmatum* L.) may also mediate interleukin-4-related immune pathways and interleukin-13 signaling. According to the immunotherapy trials of HCC in recent years [61], the checkpoint inhibitors of programmed cell death protein 1 (PD-1) and cytotoxic T-lymphocyte-associated antigen 4 (CTLA-4) engage CD8 immunity [62]. However, the core CHMs have the opposite effect and mediate CD4 cytokine signaling. Studies following the dynamics of CD8 T-cells revealed that changes in the systemic PD-1(+) CD8 subpopulation in response to PD-L1/PD-1 blockade therapy might lead to significant antitumor activities in patients [63,64], but later studies suggested that these changes may depend on systemic CD4 immunity [65,66]. A very recent review from Zuazo et al. [67] showed that the presence of specific CD4 T-cell memory subsets in peripheral blood before the initiation of treatments is a strong predictor of responses in non-small-cell lung cancer patients. Furthermore, the polysaccharides extracted from the core CHM of cluster 1, *Hedyotis diffusa* Willd, were reported to enhance the antitumor activity of cytokine-induced killer cells and could be used for cancer immunotherapy when combined with cytokine-induced killer cell therapy [68]. The extract of another core CHM of cluster 1, *Scutellaria barbata* D. Don, was reported to have anticancer effects, mediated by its immunomodulatory activity in hepatoma H22-bearing mice by downregulating Treg cells and manipulating the Th1/Th17 immune response [69]. These results not only pave the way for further studies on the pharmacological mechanism anticancer CHMs, but also provide clinical guidance for TCM practitioners to treat patients undergoing immunotherapy.

Nevertheless, our study had some limitations. First, the CGRD covers only CHM prescribed through CGMH. The HCC patients who received CHM from local clinics or other medical facilities could not be identified; hence, the use of CHM may be underestimated. Additionally, CHM is not a standard treatment for HCC, and there is no suggested timing to initiate CHM management. In clinical practice, patients autonomously ask for and consensually receive CHM management from TCM doctors; hence, accidental exposure among CHM nonusers should be minimal, and the use of landmark analysis could reduce the possibility of immortal time bias. CHM prescriptions and compositions are complicated because these prescriptions are personalized and are composed of various kinds of CHMs that are concocted on the basis of each patient’s condition. Therefore, we assessed the duration-dependent trend through exposure duration instead of a single CHM dose dependency. Furthermore, we found that the prevalence of CHM use for H-HCC was lower than CHM use among all HCC patients in Liao et al.’s report [23]. The insufficient evidence in using CHM among H-HCC patients and the inclusion of newly diagnosed H-HCC only may both contribute to the low prevalence of CHM use. Although strict inclusion criteria could help us to estimate the outcome for H-HCC more accurately, the low proportion of CHM use may lead to the issue of poor generalizability of this result. Overall, our study is still a retrospective observational study and is limited by the database used; thus, further large randomized controlled trials with specific SH or HF are still needed to confirm the actual causality of different CHM prescriptions.

## 5. Conclusions

This study indicates the feasibility of CHM use for patients with inoperable H-HCC. In addition, the evaluation of core CHMs and proposed molecular pathways disclosed the role of CHM in managing H-HCC. These results may warrant further clinical and bench studies for CHM use among patients with inoperable H-HCC.

## Figures and Tables

**Table 1:** Baseline features of patients with huge hepatocellular carcinoma (H-HCC ≥10cm). Categorical covariates are presented as counts with proportion and continuous covariates are presented as medians with interquartile range (IQR).

	All Subjects (n = 1618)	CHM Users (n = 88)	CHM Nonusers (n = 1530)	*p*
Demographics				
Gender				
Female	252 (15.6%)	18 (20.5%)	234 (15.3%)	0.19
Male	1366 (84.4%)	70 (79.5%)	1296 (84.7%)	
Age (years)	58.0 (49.0–68.0)	58.0 (50.5–66.0)	58.0 (49.0–68.0)	0.76
Age group				
<40	167 (10.3%)	11 (12.5%)	156 (10.2%)	0.75
41–60	747 (46.2%)	41 (46.6%)	706 (46.1%)	
≥61	704 (43.5%)	36 (40.9%)	668 (43.7%)	
Comorbidities				
Diabetes	288 (17.8%)	11 (12.5%)	277 (18.1%)	0.18
Hypertension	434 (26.8%)	23 (26.1%)	411 (26.9%)	0.88
Chronic hepatitis				0.73
HBV	787 (48.6%)	41 (46.6%)	746 (48.8%)	
HCV	186 (11.5%)	8 (9.1%)	178 (11.6%)	
HBV + HCV	67 (4.1%)	5 (5.7%)	62 (4.1%)	
Fatty liver	34 (2.1%)	30 (2.0%)	4 (4.5%)	0.10
Lifestyle factors				
Cigarette smoking	312 (19.3%)	11 (12.5%)	301 (19.7%)	0.097
Alcohol consumption	253 (15.6%)	14 (15.9%)	239 (15.6%)	0.94
Betel nut chewing	99 (6.1%)	2 (2.3%)	97 (6.3%)	0.12
Disease severity				
Child–Pugh Score classification				
A	658 (74.8%)	35 (68.6%)	623 (75.2%)	0.51
B	201 (22.8%)	15 (29.4%)	186 (22.4%)	
C	21 (2.4%)	1 (2.0%)	20 (2.4%)	
Cirrhosis				
No	967 (59.8%)	47 (53.4%)	920 (60.1%)	0.21
Yes	651 (40.2%)	41 (46.6%)	610 (39.9%)	
Tumor size (cm)	12.7 (11.0–15.0)	12.4 (11.0–14.0)	12.7 (11.0–15.0)	0.75
TNM staging (AJCC 8th version)				
I	73 (4.5%)	7 (8.0%)	66 (4.3%)	0.43
II	30 (1.9%)	1 (1.1%)	29 (1.9%)	
III	1057 (65.3%)	56 (63.6%)	1001 (65.4%)	
IV	458 (28.3%)	24 (27.3%)	434 (28.4%)	
BCLC classification				
B	194 (17.5%)	9 (14.8%)	186 (17.8%)	0.38
C	834 (75.4%)	50 (82.0%)	784 (75.0%)	
D	77 (7.0%)	2 (3.3%)	75 (7.2%)	
Initial HCC treatment				
TACE	437 (27.0%)	24 (27.3%)	413 (27.0%)	0.95
RFA/PEI	20 (1.2%)	0 (0.0%)	20 (1.3%)	0.28
Target therapy	397 (24.5%)	27 (30.7%)	370 (24.2%)	0.17
Chemotherapy	316 (19.5%)	14 (15.9%)	302 (19.7%)	0.38
Medications				
Anti-HCV/HBV therapy	71 (4.4%)	5 (5.7%)	66 (4.3%)	0.54
Metformin	83 (5.1%)	2 (2.3%)	81 (5.3%)	0.21
Aspirin	71 (4.4%)	4 (4.5%)	67 (4.4%)	0.94
Diuretics	129 (8.0%)	124 (8.1%)	5 (5.7%)	0.41
Biochemical profiles				
α-Fetoprotein (ng/mL)	749.7 (26.1–17,359.5)	179.2 (10.1–7700.1)	813.7 (27.5–17701.7)	0.088
Albumin (g/dL)	3.7 (3.3–4.1)	4.0 (3.5–4.2)	3.7 (3.2–4.1)	0.003
Hemoglobin (g/dL)	12.7 (11.1–14.2)	12.8 (11.2–14.1)	12.7 (11.1–14.2)	0.68
Platelet (10^3^/μL)	224.0 (157.0–302.0)	249.0 (167.5–327.5)	223.0 (157.0–299.0)	0.21
INR	1.1 (1.1–1.2)	1.1 (1.0–1.2)	1.1 (1.1–1.2)	0.21
AST (U/L)	85.0 (57.0–135.0)	80.0 (49.0–149.5)	86.0 (57.0–134.0)	0.98
ALT (U/L)	51.0 (32.0–82.0)	57.5 (35.5–85.0)	50.0 (32.0–82.0)	0.31
Total bilirubin (mg/dL)	1.0 (0.7–1.4)	0.9 (0.7–1.3)	1.0 (0.7–1.4)	0.20

Abbreviations: alanine aminotransferase, ALT; aspartate aminotransferase, AST; Chinese herbal medicine, CHM; huge hepatocellular carcinoma, H-HCC; hepatitis B virus, HBV; hepatitis C virus, HCV; international normalized ratio, INR; percutaneous ethanol injection, PEI; radiofrequency ablation, RFA; transcatheter arterial chemoembolization, TACE.

**Table 2:** Factors associated with CHM use among patients with huge hepatocellular carcinoma (H-HCC ≥ 10 cm).

	Crude OR	*p*	Adjusted OR *	*p*
Demographics				
Gender				
Male vs. Female	0.70 (0.41–1.20)	0.196	0.86 (0.36–2.05)	0.727
Age group				
>60 vs. ≤60 years	0.89 (0.58–1.38)	0.613	0.76 (0.38–1.51)	0.433
Comorbidities				
Diabetes	0.65 (0.34–1.23)	0.185	0.96 (0.35–2.64)	0.937
Hypertension	0.96 (0.59–1.57)	0.881	0.80 (0.35–1.84)	0.599
Viral hepatitis	0.88 (0.56–1.36)	0.558	0.66 (0.34–1.30)	0.233
Fatty liver	2.38 (0.82–6.91)	0.111	4.58 (1.09–19.2)	0.038
Lifestyle factors				
Cigarette smoking	0.58 (0.31–1.11)	0.101	0.36 (0.14–0.90)	0.029
Alcohol consumption	1.02 (0.57–1.84)	0.942	1.39 (0.60–3.21)	0.436
Betel nut chewing	0.34 (0.08–1.42)	0.139	0.20 (0.02–1.62)	0.131
Disease severity				
Child–Pugh Score classification				
C vs. A-B	0.87 (0.12–6.54)	0.891	1.35 (0.11–16.25)	0.812
Cirrhosis	1.32 (0.85–2.02)	0.212	2.50 (1.14–5.47)	0.022
Tumor size (per 1 cm)	0.98 (0.92–1.05)	0.582	0.99 (0.89–1.09)	0.818
TNM staging (AJCC 8th version)				
Stage III–IV vs. I–II	0.66 (0.31–1.41)	0.285	0.83 (0.2–3.41)	0.799
BCLC classification				
C vs. B	1.32 (0.64–2.73)	0.457	1.38 (0.52–3.67)	0.521
D vs. B	0.55 (0.12–2.61)	0.453	0.41 (0.03–5.28)	0.493
Initial HCC treatment				
TACE	1.01 (0.63–1.64)	0.954	0.89 (0.43–1.86)	0.764
Target therapy	1.39 (0.87–2.22)	0.170	1.76 (0.86–3.62)	0.125
Chemotherapy	0.77 (0.43–1.38)	0.379	0.59 (0.21–1.61)	0.301
Medications				
Anti-HCV/HBV therapy	1.34 (0.52–3.41)	0.544	1.94 (0.68–5.52)	0.212
Metformin	0.42 (0.10–1.72)	0.226	0.61 (0.06–6.01)	0.675
Aspirin	1.04 (0.37–2.92)	0.941	0.73 (0.08–6.46)	0.778
Diuretics	0.68 (0.27–1.72)	0.417	0.54 (0.15–1.90)	0.333
Biochemical profiles				
α-Fetoprotein (>400 vs. ≤400 ng/mL)	0.59 (0.35–0.99)	0.046	0.41 (0.21–0.81)	0.010
Albumin (≤3.5 vs. >3.5 g/dL)	0.54 (0.32–0.90)	0.017	0.50 (0.23–1.07)	0.074
Hemoglobin (≤10 vs. > 10 g/dL)	0.71 (0.34–1.51)	0.374	1.29 (0.49–3.37)	0.610
Platelet (≤100 vs. >100 × 10^3^/μL)	1.18 (0.50–2.78)	0.709	1.75 (0.51–6.00)	0.372
INR (>1.4 vs. ≤1.4)	0.39 (0.05–2.90)	0.360	1.61 (0.17–14.95)	0.673
AST (>102 vs. ≤102 U/L)	1.18 (0.75–1.86)	0.480	1.21 (0.58–2.53)	0.616
ALT (>108 vs. ≤108 U/L)	1.38 (0.80–2.41)	0.249	1.73 (0.75–4.01)	0.199
Total bilirubin (>1.5 vs. ≤1.5 mg/dL)	0.68 (0.36–1.27)	0.229	0.82 (0.34–2.01)	0.668

Abbreviations: alanine aminotransferase, ALT; aspartate aminotransferase, AST; Chinese herbal medicine, CHM; huge hepatocellular carcinoma, H-HCC; hepatitis B virus, HBV; hepatitis C virus, HCV; international normalized ratio, INR; percutaneous ethanol injection, PEI; radiofrequency ablation, RFA; transcatheter arterial chemoembolization, TACE. * Gender, age, tumor size, cancer status, cancer treatment, and feasible laboratory data were used to adjust the logistic regression model.

**Table 3:** Outcome of non-operable H-HCC.

	All Subjects (n = 1618)	CHM Users (n = 88)	CHM Nonusers (n = 1530)	*p*
Overall survival (OS)				
1 year	30.0% (27.8–32.3)	46.6% (35.9–56.6)	29.0% (26.7–31.3)	<0.001
2 year	14.8% (13.1–16.6)	27.0% (18.2–36.6)	14.1% (12.3–15.9)	<0.001
3 year	10.0% (8.5–11.5)	15.0% (8.2–23.7)	9.7% (8.2–11.3)	0.002
Liver-specific survival (%) *				
1 year	30.4% (28.0–32.9)	46.7% (35.1–57.4)	29.5% (27.0–32.0)	0.002
2 year	15.2% (13.3–17.2)	26.4% (17.0–36.8)	14.5% (12.6–16.6)	0.002
3 year	10.8% (9.2–12.6)	13.4% (6.5–22.7)	10.7% (9.0–12.5)	0.011

* Excludes 261 subjects (CHM users, n = 10; CHM nonusers, n = 251) unrelated to liver-specific mortality. Abbreviations: Chinese herbal medicine, CHM; huge hepatocellular carcinoma, H-HCC.

**Table 4:** Risk of all-cause mortality among the CHM users in relation to accumulative duration of using CHM.

	HR (95% CI)	*p*	aHR * (95% CI)	*p*
All CHM users (n = 88)	0.69 (0.56–0.85)	0.001	0.62 (0.44–0.87)	0.006
Use of CHM by accumulative duration (days)				
CHM nonusers (n = 1530)	1 (reference)		1 (reference)	
≤28 (n = 45)	0.84 (0.63–1.10)	0.204	0.85 (0.58–1.25)	0.416
>28 (n = 43)	0.54 (0.40–0.74)	<0.001	0.44 (0.26–0.74)	0.002

* Gender, age, tumor size, cancer status, cancer treatment, serum albumin level, and serum α-fetoprotein level (AFP) were used to adjust the Cox regression models.

**Table 5:** Sensitivity and subgroup analysis for overall survival estimation of CHM use.

	Unadjusted		Adjusted	
	HR (95% CI)	*p*	HR * (95% CI)	*p*
Different PS models *				
ATT (n = 1618)	0.68 (0.54–0.85)	0.001	0.69 (0.54–0.89)	0.004
IPTW (n = 1618)	0.74 (0.58–0.94)	0.014	0.76 (0.58–0.99)	0.045
Overlap weighting (n = 1618)	0.68 (0.54–0.86)	0.001	0.69 (0.54–0.89)	0.004
Different populations				
All subjects, without landmark design (n = 2331)	0.55 (0.44–0.69)	<0.001	0.58 (0.44–0.76)	<0.001
Model with 120 day landmark analysis (n = 1262)	0.70 (0.54–0.91)	0.008	0.67 (0.49–0.93)	0.016
Subgroup analysis				
Age, years				
≤60	0.75 (0.55–1.01)	0.059	0.64 (0.39–1.04)	0.069
>60	0.61 (0.42–0.89)	0.010	0.60 (0.34–1.08)	0.089
Gender				
Female	0.61 (0.36–1.02)	0.062	0.35 (0.12–1.02)	0.055
Male	0.71 (0.55–0.93)	0.011	0.72 (0.49–1.07)	0.102
Initial treatment				
No	0.62 (0.42–0.91)	0.014	0.88 (0.27–2.87)	0.831
Yes	0.73 (0.54–0.98)	0.037	0.59 (0.40–0.88)	0.009
Albumin				
>3.5 g/dL	0.84 (0.62–1.13)	0.245	0.72 (0.47–1.11)	0.135
≤3.5 g/dL	0.49 (0.30–0.81)	0.005	0.44 (0.21–0.93)	0.032
Cirrhosis				
No	0.63 (0.45–0.87)	0.005	0.44 (0.18–1.09)	0.076
Yes	0.76 (0.54–1.07)	0.114	0.69 (0.46–1.03)	0.068
α-Fetoprotein				
≤400 ng/mL	0.85 (0.59–1.23)	0.396	0.81 (0.50–1.33)	0.411
>400 ng/mL	0.59 (0.39–0.90)	0.015	0.52 (0.29–0.91)	0.022

Abbreviations: the average treatment effect for the treated, ATT; Chinese herbal medicine, CHM; hazard ratio, HR; inverse probability of treatment weighting, IPTW; propensity score, PS. * Gender, age, tumor size, cancer status, cancer treatment, serum albumin level, and serum α-fetoprotein level (AFP) were used to generate PS models and adjust the Cox regression models.

**Table 6:** The top 10 single CHM prescribed for H-HCC (prescriptions, n = 417).

CHM	Counts	Prevalence (%)
*Hedyotis diffusa* Willd.	180	43.2%
*Salvia miltiorrhiza* Bunge	132	31.7%
*Scutellaria barbata* D. Don	129	30.9%
*Astragalus mongholicus* Bunge	93	22.3%
*Curcuma longa* L.	72	17.3%
Xiang-Sha-Liu-Jun-Zi-Tang	68	16.3%
Zhen-Ren-Huo-Ming-Yin	61	14.6%
Yin-Chen-Wu-Ling-San	56	13.4%
*Paeonia lactiflora* Pall.	50	12.0%
*Rheum palmatum* L.	48	11.5%

Abbreviations: CHM, Chinese herbal medicine.

## Data Availability

The data presented in this study are available on request from the corresponding author. The data are not publicly available due to the regulations from the Institutional Review Board of the Chang Gung Medical Foundation.

## Supplementary Materials

The following supporting information can be downloaded at https://www.mdpi.com/article/10.3390/ijerph191912480/s1: Supplementary Material S1. Diagnosis codes; Supplementary Material S2. CHM combinations for CMN; Supplementary Material S3. CMN for HHCC; Supplementary Material S4. CHM.ingredients.TP.

## Abbreviations

AJCC, American Joint Committee on Cancer; BCLC, Barcelona Clinic Liver Cancer classification; CHM, Chinese herbal medicine; CMN, Chinese herbal medicine network; HCC, hepatocellular carcinoma; H-HCC, huge hepatocellular carcinoma; HBV, hepatitis B virus; HCV, hepatitis C virus; HFs, herbal formulas; OS, overall survival; PEI, percutaneous ethanol injection; RFA, radiofrequency ablation; SHs, single herbs; TACE, transcatheter arterial chemoembolization; TCM, traditional Chinese medicine.
