# Supplementary material for: The Characteristics and Mortality of Chinese Herbal Medicine Users among Newly Diagnosed Inoperable Huge Hepatocellular Carcinoma (≥10 cm) Patients: A Retrospective Cohort Study with Exploration of Core Herbs
Source: Int J Environ Res Public Health. 2022 Sep 30;19(19):12480. doi: 10.3390/ijerph191912480 (PMC9564474; doi:10.3390/ijerph191912480)
Supplement: Supplementary file 1 [file ijerph-19-12480-s001.zip › S1.Diagnosis codes.pdf]

Supplementary Table S1. Diagnosis codes used in the study

Disease	ICD-9-CM codes	ICD-10-CM codes
Hypertension	401-405	I10, I11
Diabetes	250.x, except 250.x1 and 250.x3	E11
Hepatitis B	07020-07023, 07030-70733	B169, B1910, B162, B1911, B181
Hepatitis C	07041, 07044, 07051, 07054	B1710, B1711, B182, B1920, B1921
Fatty liver	5718, 5719, 5710	K760, K7581, K700
